# Research Advances in Genetic Mechanisms of Major Cucumber Diseases Resistance

**DOI:** 10.3389/fpls.2022.862486

**Published:** 2022-05-19

**Authors:** Yujin He, Mingming Wei, Yanyan Yan, Chao Yu, Siqi Cheng, Yihan Sun, Xiangtao Zhu, Lingling Wei, Huasen Wang, Li Miao

**Affiliations:** ^1^Key Laboratory for Quality and Safety Control of Subtropical Fruits and Vegetables, Collaborative Innovation Center for Efficient and Green Production of Agriculture in Mountainous Areas of Zhejiang Province, Ministry of Agriculture and Rural Affairs, College of Horticulture Science, Zhejiang Agriculture and Forestry University, Hangzhou, China; ^2^Ministry of Agriculture Key Laboratory of Biology and Genetic Resource Utilization of Rubber Tree, State Key Laboratory Breeding Base of Cultivation and Physiology for Tropical Crops, Rubber Research Institute, Chinese Academy of Tropical Agricultural Sciences, Danzhou, China; ^3^College of Jiyang, Zhejiang Agriculture and Forestry University, Zhuji, China; ^4^Institute of Ecological Civilization, Zhejiang Agriculture and Forestry University, Hangzhou, China

**Keywords:** cucumber, powdery mildew, downy mildew, Fusarium wilt, genetic mechanism

## Abstract

Cucumber (*Cucumis sativus* L.) is an important economic vegetable crop worldwide that is susceptible to various common pathogens, including powdery mildew (PM), downy mildew (DM), and Fusarium wilt (FM). In cucumber breeding programs, identifying disease resistance and related molecular markers is generally a top priority. PM, DM, and FW are the major diseases of cucumber in China that cause severe yield losses and the genetic-based cucumber resistance against these diseases has been developed over the last decade. Still, the molecular mechanisms of cucumber disease resistance remain unclear. In this review, we summarize recent findings on the inheritance, molecular markers, and quantitative trait locus mapping of cucumber PM, DM, and FM resistance. In addition, several candidate genes, such as PM, DM, and FM resistance genes, with or without functional verification are reviewed. The data help to reveal the molecular mechanisms of cucumber disease resistance and provide exciting new opportunities for further resistance breeding.

## Introduction

Cucumber (*Cucumis sativus* L.) is a popular vegetable grown on a large scale worldwide. It has an edible fruit with refreshing tastes and is enriched with vitamin E. In the recent years, with the increasing cultivation area of cucumber, it has gradually moved towards a large-scale planting model. However, because cucumber is susceptible to horticultural diseases, including powdery mildew (PM), downy mildew (DM), Fusarium wilt (FW), Verticillium wilt, *Cladosporium cucumerinum*, Corynespora leaf spot, green mottle mosaic virus, and bacterial soft rot, it does not help for industrialized production, which results in substantial economic losses to cucumber producers. Among the diseases, PM, DM, and FW are the serious main fungal diseases of cucumber that result in severe production and quality losses (Block and Reitsma, 2005; Zhang et al., 2016; Vakalounakis and Lamprou, 2018). Several effective approaches have been widely used to control these diseases, such as various fungicides, biofungicides, and grafting. However, the variable adaptability of pathogens, fungicides residues on plants and in the environment, and higher production costs associated with these approaches indicate that better methods are required (Mahmood et al., 2016; Chen et al., 2021). Therefore, breeding more resistant cultivars is an efficient approach to control cucumber diseases and understanding the genetic and molecular mechanisms of cucumber disease resistance is a crucial focus of cucumber breeding programs.

There is no conclusive genetic data on cucumber disease resistance at present. Some studies have shown that PM, DM, and FW resistance are quantitative traits controlled by multiple genes, respectively. For instance, the resistance to PM is controlled by a recessive single gene, and susceptibility is controlled by partial dominant genes (Nie et al., 2015a). In cucumber, the DM resistance was controlled by multiple recessive genes and has the duplicate recessive epistasis and the additive effects data confirmed the detected 14 quantitative trait loci (QTLs) for DM resistance (Innark et al., 2020). Dong et al. (2019) found that the inheritance of FW resistance in cucumber is a quantitative resistance trait controlled by multiple genes, including two pairs of additive dominance-epistatic major genes and an additive-dominance polygene. However, resistance to PM, DM, and FW is also controlled by a single gene. For example, a single recessive gene, *pm*, for PM resistance in leaves, has been mapped to an approximately 468 kb region on chromosome 5 in IL52 (Zhang et al., 2018). A recessive resistance gene, *dm-1*, has been identified in many DM-resistant plant introduction (PI) lines, including PI 197087, Gy4, Chipper, and the Market more series (Barnes and Epps, 1954; Wehner and Shetty, 1997; Call et al., 2012a). *Foc* has been incorporated in the Dutch-type cucumber hybrids and has widely controlled FW of cucumber for 40 years (Vakalounakis and Fragkiadakis, 2003). Variety is a major factor in the inheritance of cucumber disease resistance. At present, the mapping population of cucumber PM-resistance genes has been mainly constructed using PI 197088, S06, WI 2757, H136, K8, and IL52. In the constructed segregation population, 19 possible PM resistance QTLs were identified (Wang et al., 2020). In the population generated using the high-resistance variety PI 197088, *pm1.1*, *pm1.3*, *pm4.3*, *pm5.1*, *pm5.3*, *pm5.4*, *pm6.2*, *pm6.3*, and *pm7.1* are the main resistance QTLs and *pm4.1* and *pm6.3* are two major QTLs in the population constructed using high-resistance variety S06. Additionally, *pm5.3* is the most important QTL for PM resistance in the population constructed using the high-resistance cultivar IL52 (Sakata et al., 2006; Liu et al., 2008; Fukino et al., 2013; Yoshioka et al., 2014; Zhang et al., 2018). Most PM-related genes are closely linked to DM-related genes; therefore, they may also play equally important roles in DM resistance (Wang et al., 2018; Zhang et al., 2018). The materials used for mapping genes associated with PM are also used for selecting genes associated with DM. At present, the DM-resistance gene mapping population has mainly been constructed using PI 197085, PI 197088, WI 7120 (PI 330628), WI 2757, S94, TH118FLM, IL52, and K8. In total, 16, 5, and 2 QTLs have been identified in PI 197085, PI 330628, and WI 2757, respectively (Wang et al., 2020). For example, Wang et al. (2018) developed 55 microsatellite markers and found that *dm5.1*, *dm5.2*, and *dm5.3* are the main resistance QTLs in the highly resistant variety PI 197088 and *dm1.1*, *dm2.1*, and *dm6.2* are the main sensitivity-related QTLs in the highly susceptible variety Coolgreen. Li et al. (2018) used 141 simple sequence repeat (SSR) markers to identify 5 QTLs, namely, *dm1.1*, *dm3.1*, *dm4.1*, and *dm5.1/dm5.2*, among which, *dm4.1* is a major resistance QTL in the cross-population derived from PI 197088 and Changchunmici. The development of resistance mechanisms against FW occurred more slowly than against PM and DM and few materials resistant to FW have been identified. Dong et al. (2019) detected a major effect QTL, *fw2.1*, in a 1.91-Mb region on chromosome 2 using F2 segregating populations derived from Superina (P1) and Rijiecheng (P2). Additionally, different identification and evaluation methods, mapping population, infection site, and pathogen races also influence the inheritance of cucumber disease resistance (Vakalounakis and Lamprou, 2018; Innark et al., 2020; Wang et al., 2020). The epigenetic variations also play important roles in crop disease resistance and are affected by environmental factors (Zhi and Chang, 2021), but the epigenetic regulation in cucumber disease resistance has not been found yet.

Many candidate genes for PM, DM, and FW disease resistance in cucumber have been identified using genetic mapping as well as transcriptomic and proteomic analyses and several genes have been cloned for functional verification. The candidate genes for these diseases are all involved in plant hormone signal transduction, cell redox homeostasis, and transcriptional regulation. In PM disease, the Mildew Resistance Locus O (*MLO*)*-like* genes, including *CsaMLO1–13*, but especially *CsMLO1*, *−8*, and *−11*, are the most studied PM genes in cucumber (Schouten et al., 2014; Nie et al., 2015b; Berg et al., 2017) and MLO-based PM resistance caused by the formation of cell wall depositions (papillae) by the plant cell directly beneath the site of PM penetration (Wolter et al., 1993), but the function is not yet unraveled. The candidate genes for DM resistance are involved in various metabolic pathways. For example, STAYGREEN (*CsSGR*), which is involved in the chlorophyll degradation pathway, plays important roles in DM disease resistance (Wang et al., 2019) and the transient expression of *CsLRK10L2*, which is a Damage-associated Molecular Pattern Molecule (DAMP) oligogalacturonan receptor and is involved in the breakdown of pectin, in *Nicotiana benthamiana* (*N. benthamiana*) leaves causes necrosis and results in high DM resistance (Berg et al., 2020). Several candidate genes of both DM and PM resistance have also been identified. The gene *Csa5M622830.1*, a GATA transcriptional factor gene, may prevent supplement nutrition from reaching DM and PM pathogens (Zhang et al., 2018). Compared with PM and DM, fewer candidate genes resist to FW have been identified. However, resistance to FW is enhanced in transgenic cucumber harboring Ginkbilobin2-1 (*GNK2-1*) (Liu et al., 2010). Additionally, a great number of candidate microRNAs (miRNAs), long non-coding RNAs (lncRNAs), proteins, and metabolites related to DM, PM, and FW in cucumber have also been identified (Li et al., 2011; Xu et al., 2019, 2021; Nie et al., 2021; Sun et al., 2021).

Although a number of molecular markers, QTLs, and candidate genes have been identified, the genetic mechanisms of cucumber disease resistance are not well understood. Here, we independently review the genetic mechanisms of cucumber resistance to PM, FW, and DM and also provide new insights into future management strategies.

## Inheritance, Quantitative Trait Loci Mapping, and Candidate Genes of Cucumber Resistance to Powdery Mildew

Powdery mildew mainly invades cotyledons, leaves, and stems, resulting in yellow, crisp dry leaves in which photosynthesis is seriously affected, thereby reducing cucumber yield. PM in cucumber is commonly caused by *Podosphaera xanthii* (*Sphaerotheca fuliginea*) and *Golovinomyces cichoracearum* (*Erysiphe cichoracearum*) (Block and Reitsma, 2005), which share the characteristics of frequent infection, short incubation period, and strong transmission. They also can occur annually during cucumber production.

### Inheritance of Powdery Mildew Resistance in Cucumber

A classical genetic analysis demonstrated that cucumber PM resistance is a quantitative trait controlled by multiple recessive genes in different germplasms (Smith, 1948; Kooistra, 1968; Morishita et al., 2003; He et al., 2013). Early in 1948, Smith (1948) suggested that PM resistance in Puerto Rico 37 was controlled by recessive genes and then, associated recessive genes were identified in the PI 2008151 and Natsufushinari varieties (Kooistra, 1968). The two recessively inherited genes linked to the QTL in chromosome 5 are responsible for PM in WI2757 (He et al., 2013). Additionally, studies have shown that PM resistance in cucumber is controlled by a single recessive gene. A single recessive gene *pm* for PM resistance in leaves has been mapped to an approximately 468 kb region on chromosome 5 in IL52 (Zhang et al., 2018). The resistance to PM in the stem of NCG-12 is also controlled by a single recessive nuclear gene (*pm-s*) (Liu et al., 2017). The recessive inheritance of PM is not convenient to use in cucumber breeding (Xu X. et al., 2016). The temperature-dependent PM resistance in PI 197088-5 is due to one recessive gene and another incompletely dominant gene (Morishita et al., 2003). Shen et al. (2011) found that cucumber PM traits are determined by the interaction of major genes and polygenes in the JIN 5-508 variety and the inheritance of major genes dominates. Xu X. et al. (2016) first reported the dominantly inherited major-effect QTL (*Pm1.1*) for PM in the Jin5-508-derived SSSL0.7 line. However, quantitative resistance under polygenic control is generally more durable than that conferred by a single dominant gene (Kelly and Vallejo, 2006). The inheritance of cucumber disease resistance is dependent on the variety and material (Wang et al., 2020) and the genetic laws governing cucumber PM resistance are still not well understood.

### Molecular Markers and Quantitative Trait Loci of Powdery Mildew Resistance in Cucumber

Effective molecular markers and QTLs controlling resistance to PM in cucumber have also been reported in recent years (de Ruiter et al., 2008; Fukino et al., 2013; He et al., 2013; Nie et al., 2015a; Wang et al., 2019). Various molecular markers have been used for mapping PM-associated loci in different cucumber species. In total, 140 PM-associated Specific-locus Amplified Fragment Sequencing (SLAFs) and two hot regions (*pm5.3* and *pm6.1*) have been identified on chromosomes 1 and 6 using an F2 segregating population derived from H136 as the susceptible parent and BK2 as the resistance donor (Zhang et al., 2015). In total, 17 SSR markers have been discovered to be linked to the *pm-s* gene, which maps to chromosome 5 between the pmSSR27 and pmSSR17 markers (Liu et al., 2017). The introgression of the 6.8-Mb segment that contains 3,016 single nucleotide polymorphisms (SNPs) causes the phenotypic variation in PM resistance between SSL508-28 and D8 (Xu et al., 2017) and this region, *pm5.1*, is consistent with major loci for PM resistance found in many studies (Nie et al., 2015a; Xu Q. et al., 2016; Wang et al., 2018). In total, 113 SNP and InDel markers significantly associated with PM resistance have been identified on chromosomes 4 and 5 using a genome-wide association analysis (GWAS) (Tan, 2021). Additionally, four QTLs (*pm1.1*, *pm2.1*, *pm5.1*, and *pm6.1*) have been identified on chromosomes 1, 2, 5, and 6 using the recombinant inbred line (RIL) population derived from a cross between PI 197088 and the susceptible line Coolgreen. Among them, *pm5.1* is the major-effect QTL, explaining 32.4% phenotypic variance, whereas the minor-effect QTL, *pm6.1*, contributed to disease susceptibility (Wang et al., 2018). Recently, *pm5.2* (30% R2 at LOD 11) and *pm6.1* (11% R2 at LOD 3.2) conferred PM resistance in an F2 population derived from a cross between PM-R (resistant) and PM-S (susceptible) (Zhang C. et al., 2021). After further studies on the segregation populations constructed from PI 197088, S06, WI 2757, H136, K8, and IL52, 19 possible QTLs for PM resistance were mapped (Wang et al., 2020). Moreover, PM resistance QTLs are also organ-dependent in cucumber. The disease indices of the hypocotyl, cotyledon, and true leaf of WI2757 were analyzed by multiple QTL mapping. *pm5.1* was the major QTL for cotyledon resistance, *pm5.2* controlled hypocotyl resistance, *pm1.1* and *pm1.2* controlled leaf resistance and both the minor QTLs, *pm3.1* and *pm4.1*, caused leaves or hypocotyls to have an increased PM susceptibility (He et al., 2013). Liu et al. (2017) showed that *pm-s*, located on chromosome 5, controls PM resistance in cucumber stem and the gene *Csa5G623470*, encoding an MLO protein, is closely related to the PM resistance of stem. Environmental factors also play important roles in the resistance to PM. Sakata et al. (2006) constructed a cucumber RIL using the PI197088-1 variety, resistant to PM, and the Santou variety, susceptible to PM, under both the high and low temperatures. Only one QTL played a role at high (26°C) and low (20°C) temperatures, which suggested that resistance was related to temperature. This was the first study on the QTL mapping of PM resistance genes at different temperatures. Like PM, DM is also an important disease in cucumber production. Many PM QTLs or genes are closely linked to DM QTLs or genes; consequently, they may also play equally important roles in DM resistance (Wang et al., 2018; Zhang et al., 2018). For example, *pm2.1*, *pm5.1*, and *pm6.1* associated with PM QTLs are colocalized with the DM QTLs *dm2.1*, *dm5.2*, and *dm6.1*, respectively (Wang et al., 2018). These studies showed inconsistent results regarding the number and locations of QTLs underlying PM and this may be due to differences in the germplasms, genetic maps, analysis methods, and environmental conditions.

### Candidate Genes or Proteins Involved in the Powdery Mildew Resistance of Cucumber

In the recent years, candidate genes or proteins associated with PM resistance have been identified using transcriptomic and proteomic analyses and genetic mapping. Differentially expressed genes (DEGs) have been identified between PM-resistant species and susceptible species, such as SSL508-28 and D8, XY09-118 and Q10, BK2 and H136, and NILs of S1003 and Near Iso-genic Lines (NIL) (*pm5.1*), using transcriptomes (Xu et al., 2017; Nie et al., 2021; Zhang P. et al., 2021; Zheng et al., 2021). These DEGs function in plant hormone signal transduction, phenylpropanoid biosynthesis, phenylalanine metabolism, ubiquinone and other terpenoid-quinone biosynthesis, endocytosis, plant–pathogen interaction, and Mitogen-activated Protein Kinases (MAPKS). In particular, genes encoding the transcriptome factors (WRKY, NAC, and TCP), peroxidase, nucleotide-binding site (NBS), glucanase, and chitinase have been analyzed (Zhang P. et al., 2021; Zheng et al., 2021). The miRNAs *Csa-miR172c-3p* and *Csa-miR395a-3p* are upregulated in PM-resistant D8 and *Csa-miR395d-3p* and *Csa-miR398b-3p* are downregulated in PM-susceptible SSSL508-28, suggesting that their target genes *AP2*, *bHLH*, *Dof*, *UGT*, and *LASPO* may play important roles in PM-inoculated cucumber leaves (Xu et al., 2020). Nie et al. (2021) showed that 49 differentially expressed lncRNAs may function as target mimics for 106 miRNAs during cucumber_PM interaction, including *miR156*, *miR159*, *miR164*, *miR166*, *miR169*, *miR171*, *miR172*, *miR6173, miR319*, *miR390, miR393*, *miR396*, and *miR5658*. Moreover, differentially regulated processes, proteins, and accumulated metabolites between different PM-resistant materials have also been detected, including flavonoid, hormones, fatty acid, diterpenoid metabolism, tetrapyrrole biosynthetic process, sulfur metabolic process, and cell redox homeostasis (Xu et al., 2019; Zhang P. et al., 2021).

A larger number of potential genes related to PM in cucumber have been identified using genetic mapping. Nie et al. (2015a) delimited the recessive major QTL *pm5.1* for PM resistance in an approximately 1.7-kb region between markers UW065021 and UW065094 and they identified an *MLO-like* gene *CsMLO1*, which encodes a cell membrane protein, as a candidate gene for PM resistance (Nie et al., 2015b). Schouten et al. (2014) obtained 13 *MLO* homologs, *CsaMLO1-13*, in cucumber. Among them, the ectopic expression of *CsMLO1* in the PM-resistant *Atmlo2-Atmlo12* double-mutant results in PM sensitivity recovery. The overexpression of *CsaMLO1* or *CsaMLO8* completely restores PM susceptibility in a tomato *mlo* mutant, whereas the overexpression of *CsaMLO11* only partially restores PM susceptibility (Nie et al., 2015b; Berg et al., 2017). To date, only *MLO* genes in cucumber have been functionally verified as being involved in PM resistance. In addition to *MLO* genes, other candidate PM resistance genes have been identified. *Csa1M064780* and *Csa1M064790*, encoding a cysteine-rich receptor-like protein kinase, are the most likely candidate PM resistance genes (Xu Q. et al., 2016). The single recessive gene *Csa5M622830*, which encodes a GATA transcriptional factor, is likely the gene for the complete PM resistance introgressed from *Cucumis hystrix* (Zhang et al., 2018). *CsGy5G015660*, which encodes a putative leucine-rich repeat receptor-like serine/threonine-protein kinase, is currently considered a strong candidate gene for PM resistance in cucumber (Liu et al., 2021; Zhang C. et al., 2021). Moreover, proteins related to PM resistance have also been identified and functionally verified. Two NBS-Leucine-rich Repeat (LRR) proteins (CsRSF1 and CsRSF2), closely correlated with Abscisic Acid (ABA) and Gibberellin (GA) signals in cucumber, are predicted to have a similar domain sequence with the *Arabidopsis* PM-resistance protein RESISTANCE TO POWDERY MILDEW8 (RPW8) (Xiao et al., 2001). The transient silencing of *CsRSF1* and *CsRSF2* reduces the resistance of cucumber to PM, whereas the transient overexpression of *CsRSF1* and *CsRSF2* improves the resistance of cucumber to PM (Wang et al., 2021). Transcription factors, such as GRAS, DNA-binding with One Finger (DoF), Eukaryotic Initiation Factor 2 (eIF2α), Polygalacturonase (PG), UDP-Glycosyltransferase (UGT), and Serine/threonine Protein Kinases (STPKs) and their target genes, are also differentially expressed after PM inoculation (Zhong, 2020). Translationally Controlled Tumor Protein (TCTP) is a highly conserved and multifunctional protein and CsTCTP1 may regulate the defense responses of cucumber or ABA signaling to control PM disease in cucumber. CsTCTP2 may regulate the Target of Rapamycin (TOR) signal in response to PM stress (Meng et al., 2018). These studies provide new insights into cucumber responses to PM and the potential genes related to PM will be highly helpful in breeding cucumber varieties with enhanced PM resistance.

## Inheritance, Quantitative Trait Loci Mapping, and Candidate Genes of Cucumber Resistance to Downy Mildew

Downy mildew of cucumber is caused by the obligate biotrophic oomycete *Pseudoperonospora cubensis*. It mainly infects leaves, but can also harm stems and inflorescences. It can occur from seedling to adult stage, but is particularly prevalent when cucumber enters the harvest stage. During the period of seedling infection, irregular chlorotic and withered yellow spots are produced on the reverse sides of cotyledons. A gray-black mold layer is produced when the plant becomes wet and cotyledons die when the infection is serious. During the adult stage, the disease gradually spreads upward from the lower leaves. At the beginning of the disease, light green water-immersion spots appear on the backs of the leaves. At the middle stage of the disease, the leaf spots fade from green to light yellow and the leaf backs become yellowish-brown. At the later stage, the disease spots converge and shrink upward from the leaf edges and finally, the whole leaf withers. In serious cases, all the leaves on the plant die (Zhang et al., 2016).

### Inheritance of Downy Mildew Resistance in Cucumber

Researchers have studied the inheritance of cucumber DM resistance. However, due to different resistance germplasms and inconsistent identification methods, there is no consensus on the genetic laws governing cucumber DM resistance. As early as 1942, DM-resistant lines were screened and DM resistance is controlled by a recessive resistance gene, *dm-1*, in many resistant PI lines, including PI 197087, Gy4, Chipper, and the Marketmore series (Jenkins, 1942; Barnes and Epps, 1954; Wehner and Shetty, 1997; Call et al., 2012a). Simultaneously, multiple recessive genes are also involved in the regulation of cucumber DM resistance in resistant germplasms, including cucumber varieties WI4783, Wisconsin SMR18, K8 and K18, PI19708, CSL0067, and CSL0139 (Doruchowski and Lakowska-Ryk, 1992; Zhang et al., 2013; Szczechura et al., 2015; Wang et al., 2016). Call et al. (2012b) identified three highly DM-resistant materials, PI 197088, PI 330628, and PI 605996, from 1,300 cucumber collections. Among them, PI 197088 is the most studied for DM resistance, with multiple genes being controlled in breeding programs (Li et al., 2018; Liu et al., 2021). PI 197088 also has high resistance to PM. There are different genetic bases of DM-resistant germplasms. Therefore, the identification of DM-associated molecular markers and QTLs in various resistant materials may help to increase the inheritance of DM through breeding programs.

### Molecular Markers and Quantitative Trait Loci of Downy Mildew Resistance in Cucumber

A variety of DM-associated QTLs has been identified in different varieties using Sequence Characterized Amplified Regions (SCAR), SSR, and SNP markers in recent years. The genetic linkage map was constructed using 66 polymorphic SSR markers and using this linkage map, 14 QTLs have been detected by evaluating DM in cotyledons as well as first and second true leaves after inoculation. LG5.1, located between the SSR03943 and SSR19172 markers, was detected at all the leaf stages (Innark et al., 2020). Based on the linkage map having 328 SSR and SNP markers, *dm4.1*, and *dm5.1*, compared with *dm2.1* and *dm6.1*, were determined to be the major effect of QTL (*R*^2^ = 15–30%) with additive effects and this has been reproducibly detected in four environments (US2013, US2014, IT2013, and NL2013) (Wang et al., 2016). In total, five QTLs associated with DM resistance have been identified on chromosomes 1, 3, 4, and 5 in seven independent experiments and *dm4.1*, explaining 27% of the phenotypic variance, has been reliably detected in all the indoor experiments (Li et al., 2018). The DM candidate QTLs related to DM have been detected using diverse evaluation methods that consist of different plant organs (cotyledons and true leaves), developmental stages (seedlings and adult plants), and evaluation criteria (lesion expansion and sporulation extent) and the *dm1.1* QTL has the largest effect on resistance among the nine QTLs detected (Yoshioka et al., 2014). In addition to QTL mapping methods, bulked segregant analyses (BSAs), next-generation sequencing (NGS), and GWASs have been the most rapid and effective ways of studying the genetic inheritance of DM resistant in cucumber. In total, five QTLs (*dm2.2*, *dm4.1*, *dm5.1*, *dm5.2*, and *dm6.1*) have been identified and *dm2.2* has the largest effect on DM resistance as assessed by combining BSA and NGS methods based on SNP markers (Win et al., 2017). Additionally, 18 QTLs have been detected through the GWAS of a core database of 97 cucumber lines, but only six QTLs (*dmG1.4*, *dmG4.1*, *dmG4.3*, *dmG5.2*, *dmG7.1*, and *dmG7.2*) are associated with stable DM resistance (Liu et al., 2021). To date, PI 197085, PI 197088, WI 7120 (PI 330628), WI 2757, S94, TH118FLM, IL52, and K8 have been used for mapping QTLs associated with DM resistance. Different cucumber germplasm resources may show stable genetic bases and QTLs for DM. For example, *dm5.1* and *dm5.2* have been detected in five resistance sources (Wang et al., 2020). New QTLs have also been detected in commonly used disease-resistant materials. In PI 197087, Berg et al. (2020) focused on a QTL on chromosome *4-DM4.1* in the NILs produced by PI 197087 and a susceptible cucumber line (HS279) and this contained three sub-QTLs: *DM4.1.1* that affects pathogen-induced necrosis, *DM4.1.2* that has additive effects on sporulation, and *DM4.1.3* that has recessive effects on chlorosis and sporulation. In general, the DM-associated QTLs varied depending on the germplasm and plant tissue as well as the developmental stage used in these analyses.

### Candidate Genes or Proteins Involved in the Downy Mildew Resistance of Cucumber

A series of candidate genes or proteins related to DM resistance have been identified in cucumber through transcriptome profiling, proteomic analysis, and fine mapping. A large number of DEGs between DM-resistant and susceptible materials were identified by transcriptome analyses and these DEGs are involved in multiple defense response-related functions, including response: hormone signaling, regulation of nutrient supply, pathogen-associated molecular pattern recognition, signal transduction, reactive oxygen species and lignin accumulation, cell cycle, protein binding and metabolism, and transcriptional regulation (Li et al., 2011; Burkhardt and Day, 2016; Gao et al., 2021). For example, five genes play important roles in the cucumber DM defense pathway: *Csa5G139760* encodes an acidic chitin endonuclease, *Csa6G080320* encodes a kinase having an LRR domain and transmembrane domain, *Csa5G471600* is a retroviral receptor-like protein, and *Csa5G544050* and *Csa5G564290* encode the RNA-dependent RNA polymerase gene (Gao et al., 2021). Consistently, differentially expressed proteins between the resistant and susceptible cucumber lines have also been identified and most of these proteins focus on cell rescue, defense, and energy metabolism (Sun et al., 2021). Zinc finger-homeodomain (ZHD) proteins encode a family of plant-specific transcription factors that are responsive to DM in cucumber, such as *CsZHD1–3*, *CsZHD6*, *CsZHD8*, and *CsZHD10* (Lai et al., 2021). Many novel QTLs for DM resistance in different cucumber species have been detected, such as *dm2.1*, *dm4.1*, *dm4.1.2*, *dm4.1.3*, *dmG2.1*, and *dmG7.1* (Win et al., 2017; Berg et al., 2020; Liu et al., 2021), and these precise molecular markers and QTLs for DM resistance are helpful for the consequent fine mapping and positional cloning of QTLs. Liu et al. (2021) identified seven DM-resistance candidate genes using GWAS, including *Csa1G575030* for *dmG1.4*, *Csa2G060360* for *dmG2.1*, *Csa4G064680* for *dmG4.1*, *Csa5G606470* for *dmG5.2*, and *Csa7G004020* for *dmG7.1*. Among them, *Csa5G606470* is a WRKY transcription factor and it was also identified within the DM-associated QTL *dm5.2* using a Bulked Sergeant Analysis with Whole-genome Resequencing (BSA-seq) analysis (Zhang et al., 2018). Cucumber *CsSGR* encodes a magnesium dechelatase and plays critical regulatory roles in the chlorophyll degradation pathway and a loss-of-susceptibility mutation of *CsSGR* results in durable broad-spectrum DM disease resistance (Wang et al., 2019). *CsLRK10L2* acts as a DAMP oligogalacturonan receptor and is involved in the breakdown of pectin, which is involved in the production of plant cell walls. This gene has been identified as a likely candidate for the sub-QTL *DM4.1.2* because the transient expression of its loss-of-function mutation *CsLRK10L2* from the DM-susceptible parent HS279 in *N. benthamiana* leaves causes necrosis (Berg et al., 2020). A series of DM- and PM-associated QTLs were also colocalized in typical Northern Chinese type cucumber K8, PI 197088, and PI 197088-derived line CS-PMR1. For example, *dm2.1/pm2.1*, *dm5.3/pm5.1*, and *dm6.2/pm6.1* have been colocated in PI 197088 (Wang et al., 2018). Several candidate genes for both the DM and PM resistance have also been identified, including *Csa5M622800.1*, *Csa5M622830.1*, and *Csa5M623490.1*. The gene *Csa5M622830.1* is a GATA transcriptional factor gene and it may prevent the nutrition from reaching DM and PM pathogens (Zhang et al., 2018). In addition, Cucumis sativus Irregular Vasculature Patterning (*CsIVP*)-RNA interference (*RNAi*) plants having higher salicylic acid levels show higher resistance to DM than wild type (WT) and it was proposed that *CsIVP* may interact with *CsNIMIN1*, which is a negative regulator in the salicylic acid-signaling pathway, to improve DM resistance in cucumber (Yan et al., 2020). At present, the candidate genes for DM resistance in cucumber identified by forward genetic analysis methods need to be verified by overexpression or knockout experiments in cucumber.

## Inheritance, Quantitative Trait Loci Mapping, and Candidate Genes of Cucumber Resistance to Fusarium Wilt

Cucumber FW, caused by *Fusarium oxysporum* f. sp. *cucumerinum* Owen (FOC), is a systemic soil-borne fungal disease and the hyphae of this pathogen penetrate cucumber roots, which causes vascular wilt. The disease causes necrotic lesions on the stem bases, foliar wilting, and eventually whole-plant wilt and even death and it occurs throughout cucumber development (Vakalounakis and Lamprou, 2018). The main factor affecting the incidence of FW is the number of FOC in the soil, which is positively correlated.

### Inheritance of Fusarium Wilt Resistance in Cucumber

To understand the genetic inheritance of FW resistance, it is important to develop resistance breeding resources and breed-resistant varieties. The inheritance of FW resistance in cucumber has been studied for a long time, but with different conclusions (Toshimitsu and Noguchi, 1975; Netzer et al., 1977; Vakalounakis, 1993, 1995; Zhang et al., 2014; Vakalounakis and Lamprou, 2018; Dong et al., 2019; Jaber et al., 2020). Dong et al. (2019) found that the inheritance of FW resistance in cucumber is a quantitative trait controlled by multiple genes using an F2 population derived from a cross between the susceptible line Superina and the resistant line Rijiecheng and several studies agreed with this inheritance of FW resistance in cucumber (Toshimitsu and Noguchi, 1975; Zhang et al., 2014). Other researchers have reported that the FW resistance in cucumber is a qualitative trait controlled by a single *Foc* gene (Netzer et al., 1977; Vakalounakis, 1993, 1995; Vakalounakis and Lamprou, 2018; Jaber et al., 2020). The *Foc* gene has been incorporated in the Dutch-type cucumber hybrids and has widely controlled FW in cucumber for 40 years (Vakalounakis and Fragkiadakis, 2003). The different patterns of FW inheritance in cucumber are also influenced by pathogen races, including races 1–3 from America, Israel, and Japan, respectively, and race 4 from China (Zhang et al., 2014). Vakalounakis and Lamprou (2018) found that *Foc* (syn.*Fcu*-1), which has been identified as a dominant FW resistance gene in the cultivars SMR-18 and WIS2757, controls FW resistance to races 1, 2, and 3, which indicates that FW resistance is not related to different pathogen races. Additionally, the *Foc* gene was found to be linked to the *Ccu* gene, which controls resistance to scab in cucumber inbred line 9110Gt, possible due to the FW and scab resistance in cucumber both being controlled by an NBS-type *R* gene (Vakalounakis, 1993; Mao et al., 2008). In the future, the availability of more natural FW-resistant resources aids in revealing the inheritance pattern of FW resistance in cucumber.

### Molecular Markers and Quantitative Trait Loci of Fusarium Wilt Resistance in Cucumber

Compared with PM and DM, there are limited reports on molecular linkage markers and QTL mapping related to the inheritance of FW resistance in cucumber. Wang (2005) identified an Amplified Fragment Length Polymorphisms (AFLP) marker E25M70 and an SSR marker CSWCT06A linked to cucumber *Foc2.1* at genetic distances of 8.12 and 5.98 cM, respectively. One major QTL, *Foc2.1*, has been screened from the F9 RILs derived from the cross between 9110Gt and 9930 and it is located between SSR03084 and SSR17631 on chromosome 2. The marker SSR17631 has been validated with an 87.88% accuracy among 46 cucumber germplasms (Zhang et al., 2014). Moreover, Zhou et al. (2015) mapped the QTL of *Foc4* resistance to FW in the region of SSR17631 and SSR00684 on chromosome 2. Another major QTL, *fw2.1*, located on chromosome 2, has also been detected and fine-mapped, with a physical distance of 0.60 Mb (InDel1248093–InDel1817308) and it contains 80 candidate genes (Dong et al., 2019). One AFLP marker of FW resistance in cucumber has been identified at a distance of 6.0 cm from the *Foc* gene and it was converted into SCE12M50_*B*_ and SCE12M50_*A*_ codominant markers (Jaber et al., 2020). The SCE12M50_*B*_ marker is located 7.0 cm away from SSR03084 and is linked to the *Ccu* locus that controls resistance to scab in cultivar SMR-18 (Mao et al., 2008; Jaber et al., 2020). Owing to the complexity of FW symptoms and the defects of related research techniques, the mechanisms and functions of these loci have not been determined and require further exploration.

### Candidate Genes or Proteins Involved in the Fusarium Wilt Resistance of Cucumber

Some FW candidate proteins and genes in cucumber have been identified using proteomic and transcriptomic analyses in different FW-resistant varieties. A comparative proteomic analysis of root proteins isolated from infected highly susceptible 995 and highly resistant F9 revealed that 15 overaccumulated proteins are mainly involved in defense and stress responses, oxidation-reduction, metabolism, transport and other processes, and jasmonic acid and redox signaling components. LRR family- and stress-related proteins may be crucial in the defense responses to FW in cucumber (Zhang et al., 2016). Moreover, defense mechanisms against oxidation and detoxification as well as carbohydrate metabolism may also be necessary for FW resistance in cucumber (Du et al., 2016). Xu et al. (2021) identified 210 and 243 differentially regulated proteins in the FW resistance Rijiecheng and high-susceptibility Superina after Foc infection. Additionally, four genes, *TMEM115* (*CsaV3_5G025750*), which encodes a transmembrane protein, *TET8* (*CsaV3_2G007840*), which functions as a tetraspanin, *TPS10* (*CsaV3_2G017980*), which encodes a terpene synthase, and *MGT2* (*CsaV3_7G006660*), which encodes a glycosyltransferase, are remarkably upregulated in both the cultivars after Foc inoculation, but with higher expression levels in Superina. In total, 14 chitinase defense-related genes have higher expression levels in FW susceptible and resistant lines and *CsChi23* may play an important role in activating a rapid immune reaction against FW (Bartholomew et al., 2019). Furthermore, other defense-related genes are activated to regulate the defense responses of cucumber to a Foc inoculation, including several genes related to ABA and ethylene (Zhou and Wu, 2009; Dong et al., 2020). *miR319a-JRL3*, *miR6300-BEE1*, *miR6300-DAHP1*, and *miR6300-PERK2* also regulate cucumber defenses against FW (Xu et al., 2021). Dong et al. (2019) identified five candidate FW-resistance genes in *fw2.1* by combining genetic mapping and a transcriptome analysis, *Csa2G007990*, which encodes calmodulin, *Csa2G009430*, which encodes a transmembrane protein, *Csa2G009440*, which encodes a serine-rich protein, and *Csa2G008780* and *Csa2G009330*, which are novel genes. This is the only report of mapping FW candidate genes in cucumber, but the functions of the candidate genes have not been verified.

## Future Prospects for Enhancing Cucumber Disease Resistance

**FIGURE 1:**
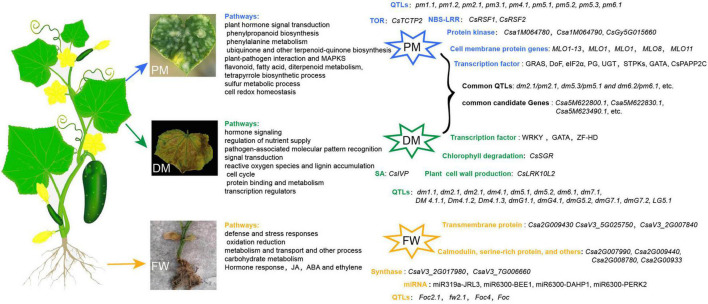
The symptoms, QTL mapping and candidate genes or proteins related to cucumber PM, DM, and FW, respectively.

In summary, the inheritance of PM, DM, and FW resistance in cucumber has been widely investigated and cucumber resistance traits are generally considered as quantitative traits controlled by more one gene. Because of the complicated inheritance of resistance to cucumber diseases, the results are not unified. Simultaneously, several molecular markers and QTLs for PM, DM, and FW resistance in cucumber have been identified (Figure 1 and Supplementary Table 1). Many factors affect cucumber resistance to these three diseases, including pathogen species, plant materials, pathogen invasion site, environment, and genetic linkage, resulting in a variety of effective molecular markers and QTLs for cucumber disease. Large numbers of candidate genes and immune proteins associated with DM, PM, and FW have been identified using mapping, GWAS, RNA sequencing (RNA-seq), and proteomic assay technology, but only a few have been functionally verified (Figure 1 and Supplementary Table 1). For example, only the functions of *MLO-like* genes that are important in PM resistance have been verified in cucumber. The transient silencing of the two NBS-LRR genes (*CsRSF1* and *CsRSF2*) reduces cucumber resistance to PM. Among the DM-resistant candidate genes, *CsSGR*, *CsLRK10L2*, and *CsIVP* have been functionally verified through mutation, transient expression, or RNAi. Additionally, the resistance of *GNK2-1* transgenic cucumber to FW is enhanced compared with WT (Liu et al., 2010). Because of a lack of cucumber FW-resistant germplasms in China, the susceptibility of most cultivars, and the relatively narrow genetic variation among cucumber FW, the breeding of cucumber FW-resistant cultivars has been restricted to a certain extent and research on the molecular mechanisms of FW has not progressed as far as research on PM and DW.

To better effectively prevent cucumber diseases and explore the genetic and molecular mechanisms of cucumber resistance to PM, DM, and FW, respectively, we propose five aspects of work that need to be performed in the future: (1) collect more disease-resistant cucumber germplasms, especially materials that are resistant to multiple pathogens, including wild germplasm resources, cultivars, and mutants; (2) identify more effective molecular markers and QTLs associated with PM, DM, and FW to be used in selecting germplasms and accelerating resistance breeding; (3) analyze more differently expressed DNA, RNA, miRNAs, lncRNAs, or metabolic related to PM, DM, or FW, respectively, through omics or multiomics and bioinformatics tools would provide considerable experimental information for mechanistic investigations and understand the regulatory network for cucumber diseases, such as transcriptomics, proteomics, metabolomics, epigenomics, and interactomics. Additionally, the data-driven interface through a user-friendly web interface would also be helpful for the mechanism of cucumber diseases, such as plant regulomics (Ran et al., 2020); (4) improve the efficiency and stability of genetic transformation in cucumber. There are now effective methods for gene functional verification that use biotechnology, such as transgenes, RNAi, Transcription Activator-like Effector Nucleases (TALENs), and CRISPR-Cas; (5) develop persistent and safe preventive measures, including chemical, biological, and physical controls. For example, maintaining an optimization of blue light in the growth light before nighttime UV is important for the management of PW in cucumber (Palma et al., 2021). A balance between effective defense and crops yield should be established through these preventive measures. The plant immunity engineering toolbox that integrates genetics, technology, and engineering is required for enhancing disease resistance in crops in the future and the molecular mechanisms of cucumber resistance to PM, DM, and FW need to be further studied.

## Author Contributions

YH, MW, and YY drafted the manuscript. CY, SC, and YS modified the manuscript. LM, HW, XZ, and LW designed the project and gave suggestions on the revision of the manuscript. All the authors approved the final version of the manuscript.

## Conflict of Interest

The authors declare that the research was conducted in the absence of any commercial or financial relationships that could be construed as a potential conflict of interest.

## Publisher’s Note

All claims expressed in this article are solely those of the authors and do not necessarily represent those of their affiliated organizations, or those of the publisher, the editors and the reviewers. Any product that may be evaluated in this article, or claim that may be made by its manufacturer, is not guaranteed or endorsed by the publisher.
