# Supplementary material for: Research Advances in Genetic Mechanisms of Major Cucumber Diseases Resistance
Source: Front Plant Sci. 2022 May 19;13:862486. doi: 10.3389/fpls.2022.862486 (PMC9161162; doi:10.3389/fpls.2022.862486)
Supplement: Supplementary file 1 [file Table_1.pdf]

Supplementary Table 1 Overview of molecular markers, QTLs and candidate genes/proteins involved in PM, DM and FW resistance in cucumber.

Disease	Causal agent	Typical symptoms	Resistance donor	Mapping Population	QTLs / candidate genes/proteins	Involved pathways	Function verification	References
Powdery mildew	*Podosphaera xanthii* (*Sphaerotheca fuliginea)* and *Golovinomyces cichoracearum (Erysiphe cichoracearum)*	mainly invades cotyledon, leaves and stems occur in the whole development period of cucumber, and the disease is serious in the middle and late stages of plant growth, resulting in yellow leaves, crisp and dry, seriously affecting the photosynthesis of cucumber leaves and	WI 2757 and True Lemon	$F_{2:3}$ populations	*Pm1.1* and *Pm1.2* for leaf resistance; *pm3.1* and *pm4.1* for leaf or hypocotyls; *pm5.1* for cotyledon, *pm5.2* for hypocotyl	-	-	He et al., 2013
			H136 and BK2	F_2 population	*pm5.3* and *pm6.1*	-	-	Zhang et al., 2015
			S1003 and S1001	F_2 population	*pm5.1*	-	-	Nie et al., 2015
			H136 and BK2	F_2 population	*Pm1.1*, *pm6.1*	defense response, toxin metabolism, cell stress response,	-	Zhang et al., 2015

	reducing cucumber yield.				and injury response		
		PI 197088 and Coolgreen	RILs population	*pm1.1*, *pm2.1*, *pm5.1*, *pm6.1*	-	-	Wang et al., 2018
		a cucumber core germplasm (CG) consisting of 94 resequenced lines	-	pmG1.1, pmG1.2, pmG2.1, pmG2.2, pmG3.1, pmG4.1, pmG4.2, pmG5.1, pmG5.2, pmG5.3, pmG5.4, pmG6.1, and *pmG6.2*	-	-	Liu et al., 2021
		SSSL0.7 and Jin5-508	F_2 population	*Pm1.1* (*Csa1M064780* and *Csa1M064790*)	cysteine-rich receptor-like protein kinase	-	Xu et al., 2016
		NCG122 and NCG121	F_2 population	*pm-s* (Csa5G623470)	encoding Mlo-related protein.	-	Liu et al., 2017

	Changchunmici and IL52	F_2 and RILs	*Pm/dm5.2*, Csa5M622830.1	encodes a GATA transcriptional factor	-	Zhang et al., 2018
	PM-R and PM-S	F_2 populations	*pm5.2* and *pm6.1, CsGy5G015660*	encoding a putative leucine-rich repeat receptor-like serine/ threonine-protei n kinase (RPK2)	-	Zhang et al., 2021
	S1003 and S05	-	*CsaMLO1*	a cell membrane protein	Ectopic expression in *Atmlo2 Atmlo12* double mutants	Nie et al., 2015
	9930, *cv. Anaxo*	no	*CsaMLO1,CsaMLO8 ,CsMLO11*	encode a plasma membrane-anch	Heterologous overexpression in the	Schouten et al., 2014; Berg et

				ored protein with seven transmembrane helices	tomato *mlo* mutant	al., 2017
	SSL508-28, Jin5-508 and D8	-	8 candidate genes underlie the *Pm5.1*(, *Csa5G569350, Csa5G606310, Csa5G600370, Csa5G600380, Csa5G604040, Csa5G606540, Csa5G606730, Csa5G512930*)	encoding receptor protein kinases, transcription factors, remorin proteins, a P-type ATPase and a 70 kDa heat shock protein.	-	Xu et al., 2017
	BK2 and H136	-	2312 DEGs	Hormone signaling, defense	-	Zhang et al., 2021

	XY09-118 and Q10	-	5478 DEGs	response,pathogen response, and peroxidase, NBS, glucanase and chitinase pathways plant hormone signal transduction, phenylpropanoid biosynthesis, plant–pathogen interaction and the MAPK signalling pathway ,	-	Zheng et al., 2021

				encoding WRKY, NAC and TCP		
	BK2 and H136	-	5832 DEGs and 971 DAMs	flavonoid, hormone, fatty acid and diterpenoid metabolisms.	-	Zhang et al., 2021
	Rijiecheng	-	miR319a-JRL3, miR6300-BEE1, miR6300-DAHP1 and miR6300-PERK2	JRL3 contains three jacalin-like lectin domains, BEE1 encoded a bHLH domain, DAHP1 encodes a 3-deoxy-D-arabino-heptulosonate	-	Xu et al., 2020

				7-phosphate synthase		
	S1003 and NIL (*Pm5.1*)	-	119 lncRNAs and 136 mRNAs	phenylpropanoid biosynthesis, phenylalanine metabolism, ubiquinone and other terpenoid-quinone biosynthesis, and endocytosis.	-	Nie et al., 2021
	D8 and SSL508-28.	-	*NRP1*(*Csa5G606550*)	nodulin-related protein 1	-	Xu et al., 2019
	XinTaiMiCi, JinYan4, B21-a-2-2-2, B21-a-2-1-2	-	Cs TCTP1, Cs TCTP2	Defense response or ABA or TOR	Transient transformation	Meng et al., 2018

						signaling pathways		
			B21-a-2-1-2 and B21-a-2-2-2	-	*CsRSF1* and *CsRSF2*	nucleotide-binding site-leucinerich repeats (NBS-LRR) proteins	transient silencing and Transient transformation	Wang et al., 2020
Downy mildew	Pseudoperonospora cubensis	mainly infects leaves and can also harm stems and inflorescences. It can occur from seedling stage to adult stage, especially when cucumber enters the harvest stage.	CS-PMR1 and Santou	RILs	*dm1.1, dm5.1* and *dm5.3*	-	-	Yoshioka et al., 2014
			PI 197087 and PI175695	F_2	*Dm1, Dm2, Dm3*	-	-	Szczechura et al., 2015
			WI7120 (PI330628) and 9930	$F_{2:3}$ population	*dm2.1, dm4.1, dm5.1* and *dm6.1*	-	-	Wang et al., 2016
			TH118FLM and WMEJ	F_2 and $F_{2:3}$	*dm2.2, dm4.1, dm5.1, dm5.2, and dm6.1*	-	-	Win et al., 2017
			PI 197088 and	F_2	*dm1.1, dm3.1,* dm4.1,	-	-	Li et al., 2018

	Changchunmici		*dm5.1, dm5.2*			
	CSL0067 and CSL0139	F_2	*Cot7_5.1_1, Cot7_5.1_2, Cot10_5.1, Cot14_5.1 First7_1.1 First7_6 , First14_5.1_1, First14_5.1_2, First14_6, Second10_6, Second14_2, Second14_5.1, Second14_6,*	-	-	Innark et al., 2020
	a cucumber core germplasm (CG) consisting of 94	-	*dmG1.4, dmG4.1, dmG4.3, dmG5.2, dmG7.1, and dmG7.2*	-	-	Liu et al., 2020

	resequenced lines					
	PI 197088 and HS279	NILs	*subQTL DM4.1.1, subQTLDM4.1.2, subQTLDM4.1.3, CsLRK10L2*	plays a role as a receptor of the DAMP oligogalacturonan, the breakdown product of pectin in the plant cell wall.	loss-of-function mutation and heterologous expression in N. benthamiana	Berg et al., 2020
	Gy14 and WI2757	$F_{2:3}$ population	*CsSGR*	in the chlorophyll degradation pathway	-	Wang et al., 2019
	Changchunmici and IL57		58 unique expressed sequence tags (ESTs)	n nearly the whole process of plant defense	-	Li et al., 2011

				such as signal transduction and cell defense, transcription, cell cycle and DNA processing, protein synthesis, protein fate, proteins with binding functions, transport, metabolism and energy		
	IL52	F_2	*Csa1G575030* for	*Csa5G606470*	-	Zhang et al., 2018

			dmG1.4, Csa2G060360 for *dmG2.1*, *Csa4G064680* for *dmG4.1, Csa5G606470* for *dmG5.2*, and *Csa7G004020* for dmG7.1	was WRKY transcription factor	
	IL53 and IL51	F_2 population	Different expressed genes	multiple functions of defense response: pathogen-associated molecular pattern recognition,	Gao et al., 2021

			signal transduction, reactive oxygen species and lignin accumulation, and transcription regulators.	
	Jinyou Sihao and - Changchun Mici	17 differentially expressed proteins	enhanced energy supply and resistance related proteins, balanced ROS production and clearance, accelerated protein	Sun et al., 2021

				metabolism and photorespiration The function of reducing photosynthesis, inducing photosystem repair and programmed cell death.		
	9930	-	13 *ZF-HD* genes(*CsMIF1-CsMIF3*, *CsZHD1-CsZHD10)*	Zinc finger-homeodomain (ZF-HD) proteins encode a family of plant-specific transcription	-	Lai et al., 2021

			PI 197088 and Vlaspik	-	different expressed of mRNA and siRNA	factors hormones and regulation of nutrient supply	-	Burkhardt and Day 2016
			R1461	-	*CsIVP*	a negative regulator in SA signaling pathway	RNAi	Yan et al., 2020
Fusarium wilt	*Fusarium oxysporum*f.sp. *cucumerinum*	a systemic infection of soil-borne fungal diseases and the hyphae of this pathogen penetrated cucumber root, which can cause vascular wilt, leads to necrotic lesions on the stem	9110Gt and 9930	F_9 RILs	*Foc2.1*	-	-	Zhang et al., 2014
			cv.SMR-18 and cv.Ashley	F_2 population	*Foc* gene	-	-	Vakalounakis and Lamprou 2018
			Superina and Rijiecheng	F_2 population	*fw2.1*	-	-	Dong et al., 2019
			Cvs Straight-8 and SMR-18	F_2 population	SCAR markers for $SCE12M50_A$ and $SCE12M50_B$ linker to *Foc*	-	-	Jaber et al., 2020

	base, foliar wilting and eventually whole plant wilt and even death, and occur throughout the growth period of cucumber	WIS2757 and Jinyan No. 2	F_2 population	*Foc-4*	-	-	Zhou et al., 2015
		Superina and Rijiecheng	F_2 population	QTL *fw2.1*	-	-	Dong et al., 2019
		Rijiecheng and Superina	-	4116 differentially expressed genes	ethylene signaling	-	Dong et al., 2020
		Rijiecheng	-	miR319a-JRL3, miR6300-BEE1, miR6300-DAHP1 and miR6300-PERK2	JRL3 contains three jacalin-like lectin domains, TaJRLL1 encodes mannosespecific jacalin-like lectin domains and regulates the salicylic acid-dependent and jasmonic acid-dependent pathways, The BEE1 gene encodes a bHLH	-	Xu et al., 2021

				domain, DAHP1 encodes a 3-deoxy-D-arabino-heptulosonate 7-phosphate synthase		
	9930	-	14 chitinase genes, Especially *CsChi23*	Pathogenesis-related proteins, and catalyzed the hydrolysis pd chitin	-	Bartholomew et al., 2019
	Jinchun No.2	-	56 differentially expressed proteins	Carbohydrate metabolism, amino acid metabolism, defense and stress responses, oxidation		Du et al., 2016

				reduction, metabolism and transport and other process	
	995 and F9	F_2 population	15 over-accumulated proteins	defense and stress responses, oxidation reduction, metabolism and transport and other process	Zhang et al., 2016
	Rijiecheng and Superina	-	32 differentially regulated proteins, three proteins with higher expression, *TMEM115* (*CsaV3_5G025750*),	MEM115 encoding a transmembrane protein, TET8 encoding function as a	Xu et al., 2021

	TET8(*CsaV3_2G007840*),*TPS10* (*CsaV3_2G017980*), *MGT2* (*CsaV3_7G006660*)	tetraspanin, TPS10 encoding a terpene synthaseand, MGT2 encoding a glycosyltransferase	
